# Burnout in UK care home staff and its effect on staff turnover: MARQUE English national care home longitudinal survey

**DOI:** 10.1093/ageing/afz118

**Published:** 2019-10-28

**Authors:** Harry Costello, Claudia Cooper, Louise Marston, Gill Livingston

**Affiliations:** Division of Psychiatry, University College London, London W1T 7NF, UK

**Keywords:** burnout, care staff, dementia, social care, older people

## Abstract

**Background:**

staff burnout and turnover lead to care home residents receiving poorer quality care. Burnout is thought to cause turnover, but this has never been investigated. We know little about which care home staffs are burnt out.

**Aims:**

to explore burnout’s relationship with staff turnover and prevalence and predictors of burnout.

**Method:**

we calculated the relationship between Maslach Burnout Inventory scores and future staff turnover (12-month number of staff leaving/number employed). We explored staff, resident and care home predictors of burnout, measured as emotional exhaustion (EE), depersonalisation (DP) and personal accomplishment (PA).

**Results:**

two-thousand sixty-two care staff in 97 care home units participated. Median yearly staff turnover was 22.7%, interquartile range (IQR) 14.0–37.7%. Care staff recorded low median burnout (median EE: 14, IQR: 7–22; DP: 1, IQR: 0–5; PA 42, IQR: 36–45). We found no association between staff burnout and turnover rate. Younger staff age was associated with higher burnout (EE coefficient − 0.09; 95% confidence interval (CI): −0.13, −0.05; DP −0.02; 95% CI: −0.04, −0.01; PA 0.05; 95% CI: 0.02, 0.08). Speaking English as a second language predicted higher EE (1.59; 95% CI: 0.32, 2.85), males had higher DP (0.02; 95% CI: 0.01, 0.04) and staff working only night shifts lower PA (−2.08; 95% CI: −4.05, −1.30).

**Conclusions:**

we found no association between care homes staff burnout level and staff turnover rates. It is a myth that burnout levels are high. Interventions for burnout could focus on at-risk groups. Future studies could consider turnover at an individual level.

## Key points

- Staff experienced an average low level of burnout but nearly a third of staff experienced high burnout.
- Younger staff age, speaking English as a second language, male staff and working night shifts predicted higher burnout.
- We found no association between care home staffs burnout level and staff turnover.

## Introduction

There are increasing numbers of people living with dementia worldwide [1]. One third of people with dementia in high-income countries live in care homes and around three quarters of care home residents have dementia [1, 2]. Staff working in long-term care facilities experience high physical and psychological workloads, which is thought to lead to burnout [3]. Higher staff burnout levels are associated with delivery of lower quality care and abusive or neglectful behaviour [4, 5].

Burnout is a work-related syndrome that describes emotional and physical exhaustion with associated negative changes in attitude towards, interest in and reward from working [6]. The most widely used measure of burnout is the Maslach Burnout Inventory (MBI), which divides burnout into three components: emotional exhaustion (EE) (a feeling of emotional depletion), depersonalisation (DP) (also described as cynicism) and negative perceptions of personal accomplishment (PA) at work [6].

In 2010, 36% of care home staff in England had been in their roles for less than 3 years, leading to calls for urgent action on high staff turnover and vacancies in dementia care in the House of Commons report on improving dementia services in England [7]. Lack of job satisfaction, high stress and burnout are suggested to lead to high turnover and vacancy rates [8–10]. No study to our knowledge has, however, investigated the relationship between turnover and burnout.

Though stress differs from burnout, in being a broader description of a state of mental strain resulting from adverse or demanding circumstances, the two are often conceptualised within the same framework [6]. Proposed descriptive models of the complex process of carer stress include the Double ABCX model [11] that focuses on the adaptation process following exposure to a crisis, and the Stress Process model [12] that identifies four domains in the process of carers adapting to events: context, stressors, resources/mediators and caregiving outcomes. However, little is known about the predictors of burnout and its role within these models.

Our systematic review and meta-analysis of burnout in care staff working in long-term care facilities found that staff caring for residents with dementia experience an average low-to-moderate burnout levels, though there may be at risk groups [13]. Preliminary evidence suggests that care home type, leadership, environment, staffing, shift-work, whether English was spoken as a second language and agitation in residents are related to care staff burnout level.

The MARQUE (Managing Agitation and Raising Quality of Life) is a naturalistic 2-year cohort study of agitation and quality of life of residents in care homes across England and included measures of staff burnout [14]. We report on the prevalence and predictors of burnout in long-term care staff and its relationship with care home staff turnover.

Our hypotheses, based on determinants of burnout identified in our systematic review [13], were that staffs are more likely to experience burnout if:

- (i) Residents had higher agitation levels
- (ii) Staff worked in a poorer physical environment
- (iii) Staffing levels were lower
- (iv) English was spoken as a second language
- (v) There was worse management support and leadership in the care home
- (vi) The care home delivered nursing level of care.

## Methods

### Setting and sample

The MARQUE study had ethical approval from the Harrow Research Ethics Committee (14/LO/0034). Care homes were recruited from across England, from January 2014 to November 2015. A care home provides residential 24-h care to those who need it. Residential care homes provide personal care and activities through care assistants and managers. Nursing homes also have qualified nurses on the staff. Care home clusters were defined as units within care homes in which staff and managers worked separately to other clusters even when within the same home. Sample size was determined by the MARQUE cohort study’s primary objective. Details of recruitment and consent procedures, including differences between participating and non-participating residents, are reported elsewhere [15].

### Procedures

We sought care home managers’ agreement for each home’s inclusion. Each manager provided a staff list and identified residents with dementia.

Care home staff asked residents, whom they judged as having decisional competence for consent for the researchers to approach them. Residents who had decisional competence for consent were asked for written, informed consent to the study. Consultees were asked to make this decision for those lacking capacity in line with the Mental Capacity Act (2005).

Trained research assistants conducted assessments by interviewing staff and residents at the care homes.

### Measures

#### Care home measures

We recorded care homes characteristics (see Table 1). We assessed the physical environment of care homes using the 15-item Therapeutic Environment Screening Survey for Nursing Homes and Residential Care (TESS), possible total scores 0–30. Higher scores indicate a better environment [16].

**Table 1:** Baseline characteristics of care homes, residents and staff.

Care home, resident & care staff measures (unless stated)			
		*n* (unless stated)	% (unless stated)
Care home type:	Nursing	58	60%
	Personal care (residential)	39	40%
Number of residents present per CH, median (IQR)		91	34 (24–50)
Number of residents with dementia present per CH, median (IQR)		91	26 (17–38)
Environmental quality score on the TESS, mean (SD)		82	16 (3)
	*n*	Mean (SD)	Median (IQR)
Turnover = (number of staff leaving CH over 12 months/number of staff at baseline) × 100	68	30.4 (23.14)	22.7% (14.0–37.7%)
MBI: emotional exhaustion	97	15.6 (4.64)	14.9 (12.3–18.0)
MBI: depersonalisation	97	2.9 (1.3)	2.6 (1.9–3.7)
MBI: personal accomplishment	97	39.2 (2.7)	39.4 (37.6–41.1)
Resident characteristics (total: *n* = 1,489)			
Resident CMAI score, median (IQR)		1,424	41 (33–55)
Resident age, mean (SD)		1,437	85 (9)
Dementia severity (CDR)	Mild	427	29%
	Moderate	482	33%
	Severe	549	38%
Staff characteristics (total: *n* = 2,062)			
Staff sex:	% Female	1,757	85.2%
Age, mean (SD)		2,002	40.0 (12.8)
Years working, mean (SD)		2,014	8.8 (8.1)
Shiftwork	Day	1,497	72.8%
	Night	164	8.0%
	Days and nights	396	19.3%
Qualifications	No qualifications	88	4.3%
	Secondary school/college	1,345	65.8%
	Degree and above	493	24.1%
	Other (diplomas)	118	5.8%
Nursing qualification	Yes	300	14.7%
	No	1,744	85.3%
English as second language	Yes	623	30.4%
	No	1,429	69.6
MBI subscale	*n*	Mean (SD)	Median (IQR)
Emotional exhaustion	2,062	15.7 (11.2)	14 (7–22)
Depersonalisation	2,062	2.9 (3.7)	1 (0–5)
Personal accomplishment	2,062	39.2 (8.5)	42 (36–45)
MBI cutoff	Emotional exhaustion	Depersonalisation	Personal accomplishment
Low burnout (*n*)	1,246 (60.4%)	1,770 (85.8%)	1,360 (66.0%)
Moderate burnout (*n*)	464 (22.5%)	242 (11.7%)	397 (19.3%)
High burnout (*n*)	352 (17.1%)	50 (2.4%)	305 (14.8%)
Number of MBI subscales high burnout experienced in	One subscale	Two subscales	Three subscales
	521 (25.3%)	75 (3.6%)	12 (0.6%)

CH: care home, SD: standard deviation.

#### Turnover

Care home managers reported the number of staff who had left in the previous 4 months at 4, 8 and 12 months after baseline. To estimate percentage yearly turnover of staff whilst accounting for care home size, the number of staff leaving over 12 months was divided by the total number of staff working at the care home over the 7 days before baseline.

The care quality commission (CQC) is an independent regulator of health and social care in England that rates care home level of care in different domains. We used the CQC standard rating to measure staffing levels and leadership/management support recording whether ‘all care standards were met’ or ‘not all care standards were met’.

### Care staff measures

We recorded sociodemographic and job characteristics of care staff (see Table 1).

Burnout was measured using the MBI a 22-item measure, comprising three subscales that record EE, DP and PA on a 7-item scale from ‘never’ to ‘everyday’ [6]. We used standard cutoff values for ‘low’ (EE: ≤16, DP: ≤6, PA: ≥39), ‘moderate’ (EE: 17–26, DP: 7–12, PA: 38–32) and ‘high’ (EE: ≥27, DP: ≥13, PA: ≤31) burnout levels [17].

### Resident measures

The staff member working most closely with a resident completed the following measures: Cohen-Mansfield Agitation Inventory (CMAI), a 29-item informant questionnaire with each item scored on a 7-item scale from ‘never’(1) to ‘several times per hour’(7) [18]. Scores >45 are regarded as clinically significant agitation [19].

Dementia severity was rated using the Clinical Dementia Rating (CDR) scale. We used the CDR standard global score based on the Washington University CDR-assignment algorithm which is a reliable and valid measure of dementia severity, categorised from 0—no dementia to 3—severe dementia [20].

### Analysis

We analysed data using Stata version 14 [21]. We first examined baseline characteristics of care homes, staff, residents and burnout.

Guided by our systematic review, we analysed predetermined independent variables and the three subscales of the MBI using mixed effects linear regression accounting for care home unit clustering. This included the following independent variables: resident agitation (mean care home CMAI score), physical environment (TESS score), staff shift type, staff age, sex, whether English was spoken as a second language, staffing level (CQC rating of staffing), management/leadership (CQC rating of management) and care home type. We explored whether number of residents with dementia, staff qualifications or number of years working might confound the relationship with burnout by analysing whether the addition of these variables caused estimates of association to differ from the unadjusted model estimate by 10% or more, though no difference was identified.

We investigated the associations between burnout and turnover at the care home level. We imputed missing data for care homes staff turnover using multiple imputation [22]. The imputation model included turnover and all variables analysed as predictors of turnover. Ten imputed datasets were created. Univariable analysis was conducted using linear regression, employing Rubin’s rules [23], of the same independent variables analysed in our burnout models and mean MBI subscale scores, with turnover as the dependent variable. Our multivariable model included burnout subscales and variables significantly associated with staff burnout or associated with care home turnover in our univariable analysis at *P* < 0.01.

## Results

### Study participants

We contacted 114 care homes, and 86 (75%) agreed to participate (see Table 1). Seven homes were subdivided into >1 cluster, totalling 18 clusters. Total baseline sample was 97 clusters. Of the 2,120 care home staff approached, 2,062 (97.3%) agreed to baseline data collection, all of whom completed the MBI.

Most care staff were female (85.2%), 30.4% spoke English as a second language, 14.7% of staff had a nursing qualification. Only 8% worked solely night shifts.

The majority of care homes were privately run (80%) and provided nursing care (60%), and there was a median of 34 residents living in each care home. The majority of residents approached had dementia (86%), and 71% of those consented had either moderate or severe dementia. Median CMAI scores across care homes was 41 (interquartile range – IQR: 33–55), and 40% of residents had clinically significant agitation.

### Turnover levels of staff in care homes

Sixty-eight of 97 care home clusters (70.1%) had sufficient data to calculate annual staff turnover (see Table 1). The others lacked complete records for the number of staff who had left in the previous 4 months at 4, 8 and 12 months after baseline. However, missing data were imputed using a multiple imputation model that included turnover and all variables analysed as predictors of turnover, and there was no difference in findings when analysis was performed with and without imputation. Median turnover was 22.7% (IQR: 14.0–37.7%) of total staff annually.

### Burnout levels in staff

On average, staff experienced low EE levels, low DP and high PA (see Table 1). However, 29.5% of all staff experienced high burnout in at least one subscale; 17.1% experienced high EE 2.4%, high DP and 14.8% had low PA (Table 1). Of staff who experienced high burnout levels, only 12.3% were burnt out in two subscales, and 2.0% in all three.

### Associations with staff turnover in care homes

We found no significant association between any burnout measure and staff turnover. Turnover of staff was significantly higher in nursing homes (see Table 2). There were no other significant determinants of turnover.

### Associations with burnout in care staff

Younger staff had significantly higher burnout levels across all three subscales (see Table 3). Male staff experienced significantly higher levels of DP. Staff who spoke English as a second language had significantly higher EE. Staff who worked only night shifts experienced significantly lower PA.

**Table 2:** Imputed univariable and multivariable regression analysis of turnover predictors in care homes

	Care home staff % turnover		
	*n*	Model 1: coef (95% CI)	Model 2: coef (95% CI)
Staff-related factors			
Mean staff emotional exhaustion	97	−0.34 (−1.74, 1.07)	−0.84 (−2.85, 1.17)
Mean staff depersonalisation	97	−0.13 (−4.15, 4.41)	1.50 (−4.01, 7.01)
Mean staff personal accomplishment	97	0.11 (−2.11, 2.32)	0.24 (−2.20, 2.68)
Staff sex: % male staff	97	0.09 (−0.40, 0.57)	−0.03 (−0.62 0.55)
Staff age	97	−0.78 (−1.79; 0.24)	−0.99 (−2.11, 0.13)
% staff that speak English as second language	97	−0.04 (−0.23, 0.15)	−0.03 (−0.24, 0.19)
Shiftwork: % staff that do night shifts	97	−0.08 (−0.34, 0.18)	−0.09 (−0.37, 0.18)
Resident-related factors			
Agitation (CMAI total)	97	−0.25 (−0.87, 0.36)	–
Care home-related factors			
CQC rating standards of staffing			
All standards met	97	Ref.	
Not all standards met		−12.31 (−43.71, 19.08)	–
CQC rating standards of management			
All standards met	97	Ref.	
Not all standards met		−4.31 (−42.21, 33.58)	–
Type of care home			
Nursing	97	Ref.	Ref.
Personal		−13.73 (−27.48, 0.03)	−16.18 (−31.90, −0.46)^*^
Average CH TESS score	97	−1.47 (−2.96, 0.02)	–

Model 1 = univariable linear regression; Model 2 = multivariable linear regression; Ref. = reference group; – = not included in multivariate analysis and CH: care home.

^*^ *P* < 0.05.

^**^ *P* < 0.001.

**Table 3:** Mixed effects univariable and multivariable analysis of burnout predictors in care staff

		Emotional exhaustion		Depersonalisation		Personal accomplishment	
	*n*	Model 1: coef (95% CI)	Model 2: coef (95% CI)	Model 1: coef (95% CI)	Model 2: coef (95% CI)	Model 1: coef (95% CI)	Model 2: coef (95% CI)
Resident-related factors							
Agitation (CMAI total)	2,055	0.03 (−0.07, 0.13)	0.01 (−0.10, 0.11)	0.001 (−0.03, 0.03)	−0.01 (−0.04, 0.03)	−0.02 (−0.09, 0.04)	−0.01 (−0.07, 0.05)
Staff-related factors							
Staff sex							
Female	1,757	Ref.	Ref.			Ref.	
Male	298	0.02 (−1.34, 1.37)	−0.41 (−1.88, 1.05)	0.83 (0.38, 1.29)^**^	0.95 (0.46, 1.44)^**^	0.01 (−1.04, 1.06)	−0.25 (−1.33, 0.83)
Staff age	2,002	−0.10 (−0.13, −0.06)^**^	−0.09 (−0.13, −0.05)^**^	−0.03 (−0.04, −0.02)^**^	−0.02 (−0.04, −0.01)^**^	0.06 (0.03, 0.09)^**^	0.05 (0.02, 0.08)^**^
English is second language							
No	1,429	Ref.	Ref.			Ref.	
Yes	623	1.46 (0.31, 2.63)^*^	1.59 (0.32, 2.85)^*^	0.28 (−0.10, 0.66)	0.28 (−0.14, 0.70)	0.17 (−0.07, 1.04)	0.85 (−0.06, 1.76)
Shiftwork							
Days only	1,497	Ref.	Ref.				
Nights only	164	−1.73 (−3.49, 0.03)	−1.52 (−3.38, 0.34)	0.02 (−0.57, 0.61)	0.07 (−0.55, 0.69)	−1.95 (−3.31, −0.58)^*^	−2.68 (−4.05, −1.30)^**^
Days and nights	396	0.31 (−0.99, 1.60)	−0.07 (−1.47, 1.33)	−0.15 (−0.58, 0.28)	−0.34 (−0.81, 0.13)	0.91 (−0.08, 1.90)	0.39 (−0.63, 1.41)
Care home-related factors							
CQC rating standards of staffing							
All standards met	1,892	Ref.	Ref.			Ref.	
Not all standards met	77	−0.50 (−5.20, 4.20)	5.81 (−2.46, 14.07)	−0.76 (−2.14, 0.61)	−0.30 (−2.88, 2.28)	−0.84 (−3.67, 1.99)	−1.63 (−6.35, 3.09)
CQC rating standards of management							
All standards met	1,907	Ref.	Ref.			Ref.	
Not all standards met	112	−2.46 (−6.33, 1.40)	−6.43 (−13.23, 0.37)	−0.66 (−1.79, −0.48)	−0.41 (−2.54, 1.71)	0.01 (−2.36, 2.37)	1.19 (−2.71, 5.10)
Type of care home							
Nursing	1,319	Ref.					
Personal	736	−0.53 (−2.43, 1.37)	−1.15 (−3.13, 0.82)	0.25 (−0.31, 0.80)	0.16 (−0.46, 0.78)	−0.52 (−1.67, 0.63)	−0.30 (−1.42, 0.83)
Average CH TESS score	1,964	−0.11 (−0.36, 0.13)	−0.04 (−0.28, 0.21)	0.02 (−0.05, 0.09)	0.02 (−0.05, 0.10)	0.04 (−0.10, 0.19)	0.04 (−0.10, 0.18)

Model 1 = mixed effects univariable analysis; Model 2 = mixed effects multivariable analysis; Ref. = reference group and CH: care home.

^*^ *P* < 0.05.

^**^ *P* < 0.001.

We found no association between mean burnout scores within care homes and turnover or any association with any of type of care home, mean agitation level within homes, CQC standard of staffing and management or quality of physical environment.

## Discussion

This is the first study to explore the relationship between staff turnover and burnout. It is also the largest survey of burnout in care staff to date. Contrary to our hypothesis and suggestions in policy documents, there was no association between the level of staff burnout in care homes and staff turnover. The low mean burnout of staff indicates that high burnout cannot be the sole reason for turnover. Additionally, there were no shared predictors of turnover and burnout suggesting that they are different phenomena. It is possible that those who find the work emotionally most difficult but stay working in care homes either become less burnt out as they become more used to the work/environment or are unable to leave. The only significant association with staff turnover was higher turnover occurring in nursing homes. Perhaps this was a result of nurses having a more portable qualification.

We found nearly a quarter of staff left the care home every year. This was high but less than the 30.7% rate estimated nationally [7]. In our cohort, 80% of care homes were privately run, and 40% were residential. This was higher than the 75% of care homes in England that were reported to be privately run and 27% that were nursing or mixed nursing and residential. When we previously corrected our results using probability weights so the homes were representative, the results were essentially unchanged [14].

Whilst turnover was measured using all staff at a care home level, we do not know whether staff who left their role and did not participate in the study experienced high burnout.

Care staff had low mean burnout levels in all domains, reflecting findings in our previous systematic review [24]. However, around a sixth of staff experienced high burnout in one domain.

All significant predictors of burnout identified were staff related; younger staff experienced higher burnout in all domains.

There has been conflicting evidence of the association between staff age and burnout in six previous studies, though this study surveyed more care staff than all of these combined [13]. It may be that older staff members who remain in the care home are resilient or have developed coping strategies.

Our finding of higher DP in male staff is similar to that in Japanese long-term care facilities [25]. It is unclear whether male staffs have a different experience of the care role to female staff. There is evidence that male care staffs experience more violence from residents [26], which is a reported predictor of burnout [27].

Staff who spoke English as a second language experienced higher EE; this is the second largest study to report this in a native English speaking country [24, 28]; the other was of Canadian care staff (*n* = 1,194) [28]. Qualitative studies of migrant care workers report common experiences of racism at work or refusal of care from staff of different ethnicity to residents [29]. Verbal abuse is linked with low morale in mental health nurses [30], and it may be that care staffs who speak English as a second language have higher burnout as a result. Additionally, it may be that the many ‘push and pull’ reasons for migration, and the differential rights of migrant care staff shaped by immigration status in both choice, and empowerment within work, influence burnout level [31].

Care staff who work nights experience lower PA likely due to the nature of contact with residents who may be asleep throughout most or all of their shift. This finding has been replicated in hospital nursing staff and midwives [32, 33].

Contrary to our hypotheses, level of resident agitation, environment quality and type of care home had no association with burnout. However, limitations of this study include the use of CQC standards of management, which are not validated measures of management or leadership, although used as national standards. Additionally, though the CMAI is a valid measure of agitation, it is not a measure of other neuropsychiatric symptoms of dementia, which were not included in our analysis due to not being identified as a determinant of burnout in our systematic review [13].

Seven care homes were subdivided into separate units. In these clusters, though staff and managers worked separately, it is likely that similar procedures and policies were shared across the whole care home which could have influenced results in these clusters.

## Conclusion

We conclude that there was no evidence of an association between staff turnover and burnout. Only care homes that delivered a nursing level of care predicted higher turnover.

It is a myth that most care home staffs have high burnout. On average, care staffs experience low burnout, and those who care for residents who are awake experience high PA in their work. However, there are a significant number of staffs who experience high burnout particularly from at risk groups. We found that predictors of higher staff burnout are focused at the individual level and largely related to sociodemographic profile. Future interventions to reduce burnout should consider targeting staffs who are younger, male, speak English as a second language and work night shifts.

**Acknowledgements**: We thank all participating care homes, residents, families and staff. We also thank all the other University College London (UCL) researchers involved in the study and members of the steering committee and community of interest.

**Author’s contributions:** The corresponding author had full access to all the data in the study and had final responsibility for the decision to submit for publication. The views expressed are those of the author(s) and not necessarily those of the National Health Service (NHS), the NIHR or the Department of Health.

**Declaration of Conflicts of Interest**: All authors declare no competing interests.

**Disclaimer**: The views expressed are those of the author(s) and not necessarily those of the NHS, the NIHR or the Department of Health.

**Declaration of Funding**: This research was funded by the UK Economic and Social Research Council and the National Institute of Health Research Grant number NIHR/ESRC ES/L001780/1. Professors Livingston and Cooper are supported by University College London Hospital (UCLH) NIHR Biomedical Research Centre and received funding from the National Institute for Health Research (NIHR) Collaboration for Leadership in Applied Health Research and Care North Thames at Bart’s Health NHS Trust. G.L. is funded as an NIHR senior investigator.

**Sponsor’s role:** The funders and sponsors of the study had no role in study design, data collection, data analysis, data interpretation, or writing of the report.
